# Risk Factors for Knee Injury in Golf: A Systematic Review

**DOI:** 10.1007/s40279-017-0780-5

**Published:** 2017-09-07

**Authors:** Matthew L. Baker, Devakar R. Epari, Silvio Lorenzetti, Mark Sayers, Urs Boutellier, William R. Taylor

**Affiliations:** 1 Institute for Biomechanics, ETH Zürich, Leopold-Ruzicka-Weg 4, 8093 Zurich, Switzerland; 2 Institute of Health and Biomedical Innovation, Queensland University of Technology, Brisbane, QLD Australia; 3 School of Health and Sports Sciences, University of the Sunshine Coast, Sippy Downs, QLD Australia

## Abstract

**Background:**

Golf is commonly considered a low-impact sport that carries little risk of injury to the knee and is generally allowed following total knee arthroplasty (TKA). Kinematic and kinetic studies of the golf swing have reported results relevant to the knee, but consensus as to the loads experienced during a swing and how the biomechanics of an individual’s technique may expose the knee to risk of injury is lacking.

**Objectives:**

Our objective was to establish (1) the prevalence of knee injury resulting from participation in golf and (2) the risk factors for knee injury from a biomechanical perspective, based on an improved understanding of the internal loading conditions and kinematics that occur in the knee from the time of addressing the ball to the end of the follow-through.

**Methods:**

A systematic literature search was conducted to determine the injury rate, kinematic patterns, loading, and muscle activity of the knee during golf.

**Results:**

A knee injury prevalence of 3–18% was established among both professional and amateur players, with no clear dependence on skill level or sex; however, older players appear at greater risk of injury. Studies reporting kinematics indicate that the lead knee is exposed to a complex series of motions involving rapid extension and large magnitudes of tibial internal rotation, conditions that may pose risks to the structures of a natural knee or TKA. To date, the loads experienced by the lead knee during a golf swing have been reported inconsistently in the literature. Compressive loads ranging from 100 to 440% bodyweight have been calculated and measured using methods including inverse dynamics analysis and instrumented knee implants. Additionally, the magnitude of loading appears to be independent of the club used.

**Conclusions:**

This review is the first to highlight the lack of consensus regarding knee loading during the golf swing and the associated risks of injury. Results from the literature suggest the lead knee is subject to a higher magnitude of stress and more demanding motions than the trail knee. Therefore, recommendations regarding return to golf following knee injury or surgical intervention should carefully consider the laterality of the injury.

**Electronic supplementary material:**

The online version of this article (doi:10.1007/s40279-017-0780-5) contains supplementary material, which is available to authorized users.

## Key Points

**Table:** 

The occurrence of knee injuries related to golf ranges from 3 to 18% of all injuries, with older players generally demonstrating a higher prevalence of injury.
The mechanisms contributing to knee injuries during golf are unknown, but reports from the literature suggest that high joint loading and complex motions may increase risk of injury, especially in the lead (target-side) knee.
Clinicians, coaches, and players alike should carefully consider participation in or return to golf when knee pain is present or following knee injury or surgical procedures (including total knee arthroplasty), especially when the lead knee is of concern.

## Introduction

Golf is considered a low-impact sport, resulting in the common perception that only low loads and stresses are placed upon the body and that players are subject to only a minor risk of injury. Many golfers enjoy the sport recreationally as a low-impact form of exercise that also plays an important part in participants’ social lives. These factors mean golf is a popular sport for older generations, as well as an activity often recommended following lower limb joint arthroplasty [1–4]. However, chronic and acute injuries are commonly reported in golf, with the lower back and the knee accounting for the majority of chronic injuries [5]. In fact, injuries to the knee are thought to account for up to 18% of all injuries in golf [6]. Despite this, a major challenge in the accurate acquisition and classification of lower limb musculoskeletal injury data is attributing their occurrence purely to involvement in golf. Furthermore, most studies reporting injuries in golf have been based on data from retrospective and self-reported surveys that were not sufficiently specific with regard to anatomical location and mechanism of injury [6]. However, even with lack of specificity, there does appear to be a general consensus suggesting the most likely causes for golf-related injuries are associated with poor or inconsistent technique and overuse as a result of repetitive training [6].

A commonly cited study assessing loading conditions in the knee suggested that the mean peak compressive forces do not exceed 99% of body weight (%BW) in either knee [7]. These findings were the foundations of a subsequent review on risk factors and mechanisms of knee injuries in golfers [8] that suggested these magnitudes of joint force are insufficient to cause damage to the knee ligaments. As a result, the risk of knee injury during golfing was concluded to be minor, but this appears to contradict previous epidemiological data. Compared with other activities such as level walking, where internal tibio-femoral joint contact forces of 267%BW have been measured in vivo [9], the loading estimations of Gatt et al. [7] seem to be exceedingly low and may underestimate the real forces that occur in the joint. Indeed, in vivo assessments of tibio-femoral joint contact forces during the golf swing in subjects who possessed an instrumented total knee arthroplasty (TKA) found compressive loads of 320–440%BW in the lead knee and 320%BW in the trailing knee [10, 11]. Whilst the low forces reported by Gatt et al. [7] are entirely possible, it seems that the disparity in magnitude arises from the fact that these authors actually reported the resultant external forces and moments from an inverse dynamics approach rather than the internal joint contact forces that include the contribution of all muscles and ligaments crossing the joint. As a result, the contribution of knee joint loading as a risk factor for injury in golf assessed in previous reviews [8] may have been considerably underestimated given the conclusions were based on these findings.

The differences in these modelling and in vivo results suggest the soft tissue structures indeed play a key role in producing potentially high forces in the knees during the golf swing, a critical aspect that is not considered in inverse dynamics models. As a result, it seems that the current interpretation of the literature regarding the prevalence of injury among both amateur and professional golfers, the loads that act in the knee during golf, and the likelihood of these loads causing injury is somewhat misleading. The aims of this systematic review were to establish (1) the prevalence of knee injury resulting from participation in golf and (2) the risk factors for knee injury from a biomechanical perspective, based upon an improved understanding of the internal loading conditions and kinematics that occur in the knee from the time of addressing the ball to the end of the follow-through.

## Methods

### Database Search and Selection Criteria

In January 2016, a systematic literature review was conducted to identify studies reporting results relevant to knee injuries and knee biomechanics during golf. A search string was constructed that combined the term “golf” using an AND operator with keywords (in truncated form combined by means of the OR operator) including “injury,” “force,” “load,” “moment,” “kinematic,” “electromyography,” and “arthroplasty.” The search was constrained to English-language, peer-reviewed articles, titles, abstracts, and keywords. The search string was used to search the following four databases; Scopus, ISI Web of Science, EMBASE, and PubMed.

**Fig. 1:**
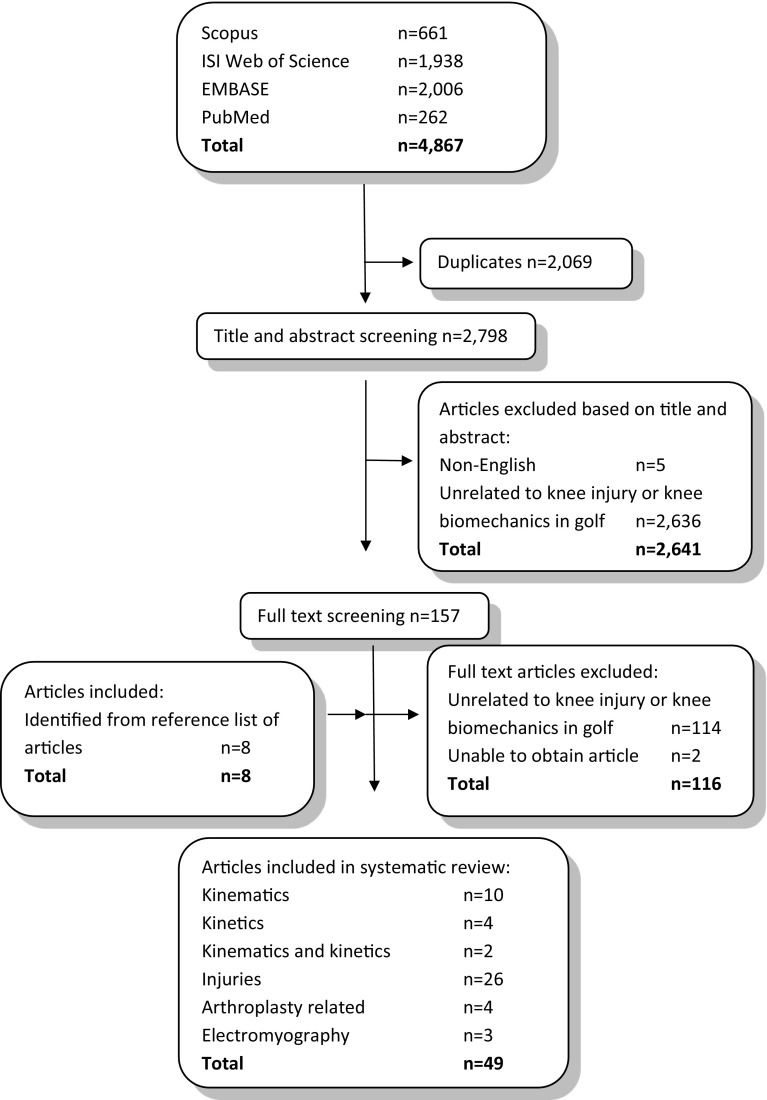
Search strategy used to acquire articles for this systematic review

The combined database search returned 4867 results, from which 2069 duplicates were removed (Fig. 1). The titles and abstracts of the remaining 2798 sources were then screened manually, and inclusion criteria dictated that only English-language peer-reviewed articles reporting injuries or biomechanics of the knee associated with golf were included. The full text and reference lists of the remaining 157 articles were screened, and 41 articles were found to meet all inclusion criteria. An additional eight sources were retrieved from the reference lists, resulting in 49 studies incorporating injury (*n* = 30) and biomechanics (*n* = 19) data.

### Data Extraction, Synthesis and Analysis

It was necessary to normalize the extracted data from all studies to allow for inter-study comparisons. Studies reporting both internal and external forces occurring at the knee were converted to %BW, whereas moments were converted into normalized moment (Nm) per kg. Equation 1 was used to account for the propagation of error $\sigma_{M}$ inherent when normalizing moments reported with a mean (*A*) and standard deviation ($\sigma_{A} )$using subject parameters such as body weight and height, both of which are also measures with associated means (*B*) and standard deviations $(\sigma_{B} )$.

1 $$\sigma_{M} \approx \left| M \right|\sqrt {\left( {\frac{{\sigma_{A} }}{A}} \right)^{2} + \left( {\frac{{\sigma_{B} }}{B}} \right)^{2} } ,$$

where $$M = {\text{mean normalized moment}}\; ({\text{Nm}}/{\text{kg}}).$$

Studies reporting knee flexion angles (*α*, in °) were combined to produce group (skill level and club type) means and standard deviations ($\sigma_{\alpha }$) throughout the swing, according to the methods for combining groups suggested by the Cochrane Collaboration [12] (Eqs. 2, 3). The method was applied sequentially in instances where two or more subject groups were combined, with $n_{1}$ and $n_{2}$ participants, respectively.

2 $$\alpha = \frac{{n_{1} \alpha_{1} + n_{2} \alpha_{2} }}{{n_{1} + n_{2} }}$$

3 $$\sigma_{\alpha } = \sqrt {\frac{{(n_{1} - 1)\sigma_{1}^{2} + (n_{2} - 1)\sigma_{2}^{2} + \frac{{n_{1} n_{2} }}{{n_{1} + n_{2} }}(\alpha_{1}^{2} + \alpha_{2}^{2} - 2\alpha_{1} \alpha_{2} )}}{{(n_{1} + n_{2} - 1)}}} .$$

To standardize the description of kinematic timing, the 12 phases of the golf swing reported across all studies were condensed into six major phases: address, top of the backswing (top-BS), middle of the downswing (mid-DS), impact, middle of the follow-through (mid-FT), and end of the follow-through (end-FT).

## Results

### Injuries

Of the 30 articles that reported injuries to the knee, 22 were surveys that considered amateur and/or professional golfers, either independently of their knee condition (*n* = 18) or specifically after knee arthroplasty (*n* = 4). Additionally, eight articles presented case studies where golf was identified as a contributing factor to knee injury.

#### Injuries Independent of Knee Condition

The rate of knee injury varied from 3 to 18% of the survey population (Table 1), but little information was reported on the nature of the injury or which knee was affected. Five studies reported career injury rates among professionals ranging from 5.5 to 15% [5, 13–16], and 11 studies reported an injury rate of 3.2–18.9% for amateur golfers [5, 17–26], with one study suggesting a generally higher injury rate in professionals compared with amateurs (5.5 vs. 3.2%) [5]. Conversely, two studies indicated that less skilled players (players with a higher handicap) may be more prone to knee injury [17, 27]. Three of the five studies comparing male and female golfers showed a higher rate of knee injuries among men [14, 17, 18], whereas equivalent injury rates were observed in male and female players in professional golf groups [16].

**Table 1:** Prevalence of knee injury in golf, population characteristics and potential injury mechanisms extracted from injury surveys (*n* = 18) obtained through a systematic search of the literature

References	Cohort	Study type	General injury reports	Knee injury reports
Batt [17]	Amateur golfers: male *n* = 164, age 49.5 y (17–85), HC 14.2 (2–24); female *n* = 29, age 53 y (27–83), HC 23.4 (5–36)	Retrospective survey; period unspecified	57% of players reported an incidental or actual injury: 72 acute injuries (while playing golf), 82 chronic injuries (aggravated by playing golf)	Actual injuries: men *n* = 4 (8%), women *n* = 0. Incidental injuries: men *n* = 8 (12%), women *n* = 2 (12%). Mean age for knee injury during play 35.6 y. Mean age for chronic knee injury 54.6 y. Mean HC of players with knee injury 17.3. Cause for knee injury obtained during play: incorrect swing/mishit 2, uneven ground 2
Dhillon et al. [18]	Amateur golfers: male *n* = 200, female *n* = 40, mean age of injured players 51 y	Retrospective verbal interview; period: entire career	193 total injuries. Injury per HC bracket: 0–9 (61.8%), 10–17 (51.8%), 18–36 (36%)	Knee injuries: male *n* = 18 (18.9%), female *n* = 1 (6%), total *n* = 19 (17%)
Finch et al. [26] (in Cabri et al. [6])	Amateur golfers, median age 40.5 y (24–65 y)	Unspecified		Knee injuries total: 18%
Fradkin et al. [19]	Amateur golfers: female *n* = 522, median age 54 y (16–75), median HC 17 (2–44)	Retrospective survey; period: previous 12 months	184 injuries: 38.6% reported recurring injury. Self-reported injury mechanism: overuse (43.6%), technical error (18%)	Knee injuries total *n* = 13 (7%). Median age of knee injury group 62 y (highest median age of all groups per injury location)
Fradkin et al. [27]	Amateur golfers *n* = 547; male 75.9%, female 24.1%	Retrospective assessment of hospital records	Presentations to hospital ED: 10.8% required hospitalization	Knee injuries total *n* = 4 (4.4%). Golfers aged > 65 y had the highest rate of lower-limb injuries
Fradkin et al. [28]	Amateur golfers *n* = 304; male 71.4%, female 28.6%, median age 53 y, median HC 13	Retrospective survey; period: previous 12 months	36.5% of subjects reported 111 injuries: 51.4% of injuries required treatment. Self-reported injury mechanism: overuse (29.7%), overexertion (26.1%)	Knee injuries *n* = 8 (7%). Median age of knee injury group: 66 y (highest median age of all groups per injury location). Median HC for knee injury group: 16.5
Gosheger et al. [5]	Amateur golfers, HC 21.5 ± 14.7, male *n* = 456, female *n* = 187; professional golfers, male *n* = 54, female *n* = 6, mean age 46.2 ± 17.3 y	Retrospective survey; period: entire career	Amateur injury rate 39.7%, professional injury rate 60%; HC did not affect injury rate in amateurs	Knee injuries: amateur *n* = 17 (3.2%), professional *n* = 6 (5.5%), total *n* = 23 (3.6%). Self-reported overuse knee injury 95.7%. Knee injury symptoms longer than 1 y, *n* = 7 (30.4%); previous chronic knee injury: 9.5%
Guten [29]	Amateur and professional golfers, 35 right-handed, male *n* = 28, female *n* = 7, mean age all players 56 y (21–73), HC 18 (0–48)	2-y case history of reports to a knee clinic		Right knee *n* = 17, left knee *n* = 15, bilateral knee *n* = 3. Type of injury: medial meniscus *n* = 17, osteoarthritis *n* = 10, lateral meniscus *n* = 4, patella chondromalacia *n* = 2, loose bodies *n* = 2, total = 35
Hadden et al. [13]	88 professional golfers	Consultation records from British Open physiotherapy service; period: 7 y	88 injuries reported	Knee injuries total: 7%
McCarroll and Gioe [14]	Professional golfers, male *n* = 127, age 30 y (23–72); female *n* = 99, age 24 y (22–42)	Retrospective survey; period: entire career	103 men reported 192 injuries; 87 women reported 201 injuries; average of two injuries per player; injuries due to repetitive practice swings 68.7%; injuries occurring during competition 7.3%	Knee injuries: in men *n* = 14 (7.3%), in women *n* = 12 (6%), total *n* = 26 (6.6%). Subjective reports of knee injury timing: impact phase 30.4%, follow- through phase 38.5%
McCarroll et al. [21]	Amateur golfers, age 52 y (15–86); male *n* = 942, HC 14; female *n* = 202, HC: 35	Retrospective survey; period unspecified	708 (62%) of those surveyed reported 908 injuries; 1.28 injuries per golfer; male injuries *n* = 584 (62%); female injuries *n* = 124 (61%); common self-reported cause of injury: excessive play or practice *n* = 204; poor swing mechanics *n* = 150; HC associated with injury rate: < 1–9 (67.5%), 10–17 (61.8%), > 17 (59.0%)	Knee injuries: in men *n* = 52 (8.9%), in women *n* = 14 (11.3%); total *n* = 66 (9.3%)
McHardy et al. [22]	Amateur golfers: male *n* = 1316, age 54.3 ± 15.3 y, HC 18.1 ± 7.0; female *n* = 318, age 59.2 ± 12.2 y, HC 26.3 ± 9.5	Retrospective survey; period: previous 12 months	288 subjects reported one or more injuries; average injury rate: 17.6%. Self-reported injury timing: follow-through 30.2%, downswing 17.7%; common self-reported injury mechanism: incorrect swing/poor technique 44.8%, overuse 25.3%; 57.3% of injuries occurred over extended periods	Data extracted manually from publication graphics. Knee injuries total: 8.3%
McHardy et al. [23]	Amateur golfers: male *n* = 473, age 58.7 ± 13.5 y, HC 17.8 ± 6.5; female *n* = 115, age 60.8 ± 9.9 y, HC 26.8 ± 9.2	Prospective survey; period: previous 12 months	78 players reported 93 injuries. Self-reported injury mechanism: swing technique 46.2%, overuse 23.7%. Self-reported injury timing: impact 23.7%, follow-through 21.5%, slow onset 13%, downswing 7.5%	Data extracted manually from publication graphics. Knee injuries total: 8.7%
McNicholas et al. [30] (in Cabri et al. [6])	286 amateur and professional golfers, age range 0–70 y	Unspecified		Knee injuries total: 13%
Smith and Hillman [15]	Professional golfers: European PGA tour players	Consultation records from tour physiotherapy van; period: 2 y, 2005, 2006 (36 tournaments)	2328 consultations were considered to be related to injury. Joint and muscular conditions were the most common injury type (92.7%)	Knee injuries total *n* = 78 (3.4%); 2005 *n* = 22 (2.1%); 2006 *n* = 56 (4.3%)
Stude et al. [24]	Amateur golfers: male *n* = 322, female *n* = 79	Retrospective survey; period: entire career	12% reported being injured playing golf; 74% reported pain or discomfort subsequent to playing golf; 35% of those with pain believed it interfered with their ability to play	8% of pain reports were localized to the knee
Sugaya et al. [16]	Professional golfers: male tour golfers *n* = 115, age 35 y (21–54); male senior tour golfers *n* = 55, age 53 y (50–63); female tour golfers *n* = 113, age 31 y (20–48)	Retrospective survey; period unspecified	Total injuries *n* = 458; male tour *n* = 203; senior male tour *n* = 102; female tour *n* = 153	Total knee injuries *n* = 26 (9%); male tour *n* = 9 (8%); senior male tour *n* = 8 (15%); female tour *n* = 9 (8%)
Thériault et al. [31] (in Thériault and Lachance [25])	Amateur golfers: male *n* = 378; female *n* = 217; age range 12–70 y	Retrospective survey; period unspecified	Male injury rate 23.3%; female injury rate 29.0%; total injury rate 25.2%, 1.31 injuries per golfer. Self-reported injury mechanism: overuse 20%, technical errors/deficiencies 62.7%. Injury type: sustained over prolonged period 54.5%, single trauma 45.5%	Total knee injuries 4%

Values are presented as mean ± standard deviation (range) unless otherwise indicated

*ED* emergency department, *HC* handicap, *PGA* Professional Golfers’ Association, y year

Many surveys questioned participants regarding the mechanisms and timing of their golfing injury, but only limited data refer specifically to the knee. Players surveyed by Batt [17] attributed their knee injury to an incorrect swing/mis-hit or standing on uneven ground (50%, respectively). Gosheger et al. [5] reported that 95.7% of players felt their knee injury was due to overuse. Additionally, McCarroll and Gioe [14] reported that the impact (30.4%) and follow-through (38.5%) phases of swing were the common time points of injury.

In all three surveys conducted by Fradkin et al. [19, 27, 28], the median age of golfers was consistently highest in those experiencing injuries at the knee, with one survey revealing that players aged >65 years were at greatest risk of lower limb injuries. Sugaya et al. [16] presented similar findings: senior male professional tour players experienced knee injuries at a higher rate (15%) than regular male (8%) and female (8%) professionals. However, in a cohort of golfers with a mean age of 50 years, Batt [17] found the average age of players with knee injuries was 35.6 years. One study that specifically addressed the issue of knee injuries in right-handed golfers found that the distribution between left (15) and right (17) knees was comparable (only three bilateral) [29]. Since this was the only formal survey reporting on the laterality of a player’s injury, it remains difficult to establish which knee is injured more often.

#### Arthroplasty-Related Injuries

Orthopedic surgeons commonly recommend golf as a rehabilitative activity following TKA, independent of whether the TKA involved the lead or the trailing knee [1–4]. In their survey of active amateur golf players after TKA (96% right-handed players, minimum 3 years post-operative), Mallon and Callaghan [3] found that 15.7% of subjects experienced a mild ache while playing golf and 34.9% experienced aching pain after playing (Table 2). Additionally, there was a statistically significant higher rate of pain during and after play in patients who received left knee TKAs (almost entirely lead knees). Another significant finding was that 54% of all TKAs and 79% of those with cemented implants had experienced radiographic loosening since their procedure (Table 2) [3]. In a separate study, three professionals and 39 amateur golfers with TKAs were surveyed following return to golf. No professionals reported any pain, injury, or revision at the mean follow-up of 4 years [32]. Only 10% of the amateur players reported pain (mean 5 years post-operatively), which was lower than the preoperative levels. Finally, when comparing TKA golfers and age-matched control players, TKA players have been found to experience higher rates of mild and considerable pain (TKA: 49 and 6% vs. controls 7 and 0%, respectively) [33].

**Table 2:** Prevalence of knee injury and/or pain in total knee arthroplasty golfers, population characteristics and potential injury mechanisms extracted from injury surveys (*n* = 4) obtained through a systematic search of the literature

Study	Cohort	Study type	Participation and surgeon advice	Knee injury and pain reports
Mallon and Callaghan [3]	Amateur golfers, 83 TKA pts: 62 men, 21 women, 47 left TKA, 26 right TKA; mean age at follow-up 65.4 y; 80 golfers were right handed	Retrospective survey; follow-up period: minimum of 3 y post-operative; radiographs obtained for 54 subjects	78.3% were recommended to use a cart; 86.7% used a golf cart after TKA; 75.9% found no shot harder after TKA; 16.9% found every shot harder after TKA	Right TKA: pain during play 8.3%, pain after play 25%. Left TKA: pain during play: 21.3%, pain after play 42.6%. Indication of radiographic loosening in 53.7% of all prostheses, 79.1% of cemented prostheses, 44.5% of uncemented prostheses
Mallon et al. [32]	Amateur *n* = 39, age at surgery 63 y (49–78); professional *n* = 3, age at surgery 58 y (52–65)	Retrospective survey: professional follow-up: 4 y (2–8) post-operative; amateur follow-up: 5 y (0.5–11) post-operative	Professionals: All were able to continue play and teaching post TKA. Amateurs: All continued to play at least 3 times per week	Professionals: no injury reports, no revisions at time of survey. Amateurs: 90% had no discomfort during play, 10% had some pain but less than pre-operative levels, one revision from the cohort
Noble et al. [33]	TKA pts: 105 women aged 71 ± 11 y, 71 men aged 70 ± 9 y	Retrospective knee function survey of TKA golfers vs. results from age-matched control golfers		Control subjects: significant pain 0%, some pain 7%. TKA subjects: significant pain 6%, some pain 49%
Jackson et al. [72]	Amateur golfers, 93 TKA patients: 80% male, 20% female, mean age at TKA 66 y (44–79), HC 11–30; right TKA 36 (39%); left TKA 17 (18%); bilateral TKA 40 (43%); 85 (91%) were right-handed golfers	Retrospective survey; follow-up period: mean 8.7 y (6.4–12.1) post-operative	91% had played golf for ≥10 y. Rounds per month: 33% less than one, 36% 2–7; 31% ≥ 8. 30% received surgeon advice. Of these, 59% were restricted to using a golf cart, 30% were restricted to spike-less shoes, 15% received swing advice from surgeons, 86% made no swing changes post TKA	83% had less pain post TKA, 13% had more pain post TKA, 28% felt driving the ball was easier post TKA, 20% found driving the ball and bunker play harder post TKA

Values are presented as mean ± standard deviation (range) unless otherwise indicated

*HC* handicap, *pt(s)* patient(s), *TKA* total knee arthroplasty, *y* year

#### Case Reports

While most studies have failed to report the type of knee injuries, case studies offer some indication as to the structures that are susceptible to damage when playing golf. Fractures and osteochondral fractures of the patella, tibial stress fractures, failure of polyethylene knee arthroplasty components, and medial meniscal tear, have all been reported while playing golf [29, 34–38]. Interestingly, medial meniscus tears have been reported as the most commonly diagnosed injury (17 of 35 injuries) followed by joint degradation due to osteoarthritis (10 of 35) [29] (Table 1).

### Kinematics

In total, 12 studies have reported on knee kinematics during the golf swing [39–50]. Kinematics were measured using retroreflective markers and infrared camera systems in all studies except that of Hamai et al. [39], which employed high frame-rate continuous X-ray imaging. These 12 reports yielded only a small representative population, with large variances in sample size and little consistency in subject selection. These factors made comparisons between groups and assessment of influences on kinematic differences difficult to establish. As a result, only two major comparisons were made: kinematic differences between skill levels according to handicap (HC), and kinematic differences between different clubs. Players were categorised into three skill groups: amateurs (HC >10), skilled amateurs (HC 1–10), and professionals (HC 0). Club types were also classified into three groups: driver, mid-irons (5–7), and pitching wedge or 9-iron.

The majority of reports in the literature address motion of the knee about the flexion–extension axis, but only two studies included kinematics regarding internal/external rotation. No studies reporting tibio-femoral abduction/adduction or translations in any plane during the golf swing were found as a result of this literature review.

#### Lead Knee Flexion/Extension

**Fig. 2:**
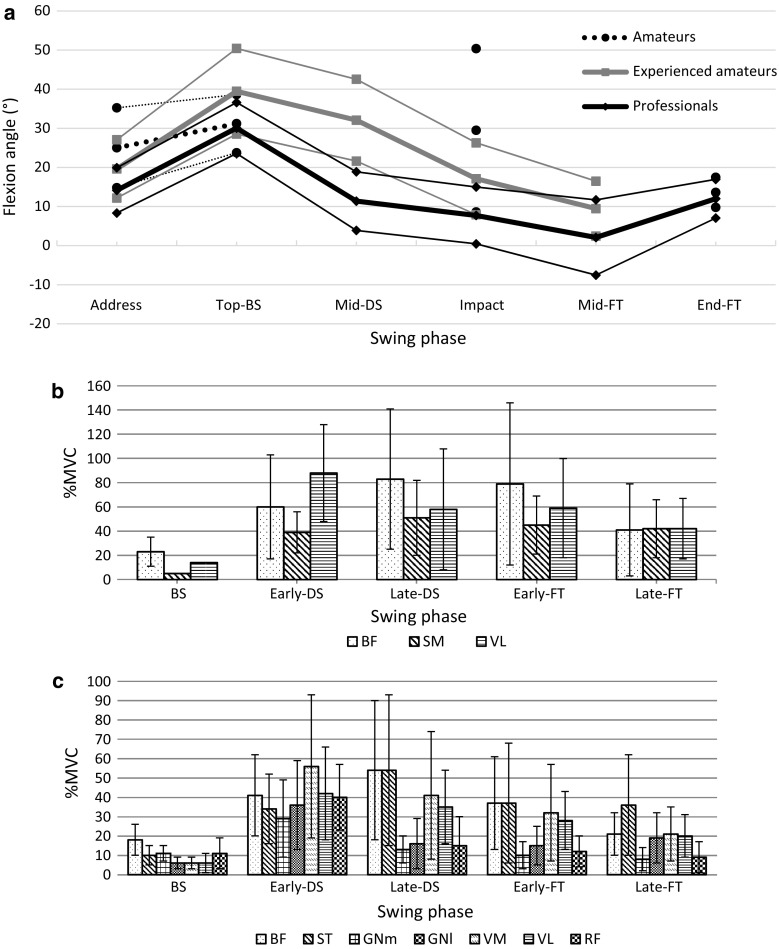
**a** Lead knee flexion angle during swings of amateur (HC >10), skilled amateur (HC 1–10), and professional golfers using all club types. Where the description of two phases between studies was similar, the respective data were merged into a common phase when at least five studies had reported results. This resulted in the establishment of six major phases throughout the swing: address, top of the backswing (top-BS), middle of the downswing (mid-DS), impact, middle of the follow-through (mid-FT), and End-FT. Thick lines and thin lines represent the combined mean and standard deviation of all study groups, respectively [39–50]. Electromyographic activity (mean ± standard deviation) as a percentage of muscle activity during maximum voluntary contraction (%MVC) of muscles crossing the knee joint at five phases of the golf swing: BS, early-DS, late-DS, early-FT, and late-FT, reported by **b** Bechler et al. [53] and **c** Marta et al. [54]. Muscles analysed: biceps femoris (BF), semimembranosus (SM), vastus lateralis (VL), semitendinosus (ST), gastrocnemius medialis (GNm), gastrocnemius lateralis (GNl), vastus medialis (VM), rectus femoris (RF). *HC* handicap

**Fig. 3:**
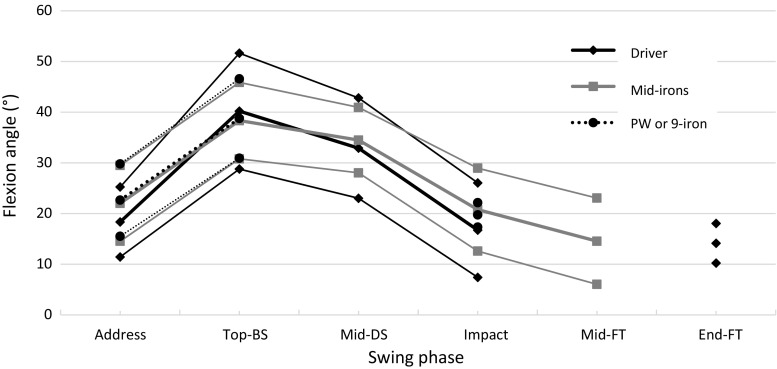
Lead knee flexion angle during swings of players of all skill levels using a driver, mid-iron (5–7), and pitching wedge (PW) or 9-iron during the six major phases of the swing: address, top of the backswing (top-BS), middle of the downswing (mid-DS), impact, middle of the follow-through (mid-FT), and end of the FT (End-FT). Thick lines and thin lines represent the combined mean and standard deviation of all study groups, respectively [39–50]

Knee flexion angle in the lead knee at the top-BS varied only little across most studies according to both club and skill level of the player (Figs. 2, 3). However, our understanding of flexion angle at impact remains less clear. Here, Somjarod et al. [50] observed noticeable differences between professional (*n* = 2) and amateur (*n* = 2) players, but no such differences were observed in another study assessing a cohort of 308 players of differing skill levels [43]. Additionally, no significant differences in flexion angles were observed during any swing phase when kinematics were measured during swings using either a driver, 5-iron, or pitching wedge [40]. Similar patterns of rapid extension were observed during the mid-DS phase in professional (234 ± 24 deg/s) and amateur (184 ± 30 deg/s) cohorts [50]. However, players achieving a high ball velocity have been shown to have significantly higher rates of lead knee extension than players with a low ball velocity at both the early- and mid-DS phases (164 ± 62 vs. 53 ± 69 and 238 ± 76 vs. 177 ± 47 deg/s, respectively) [46].

Despite the aforementioned similarities in kinematics with respect to skill level and club, one study indicated sex may indeed play a role. Egret et al. [42] found that males experienced greater flexion in the lead knee at the top-BS (35° ± 5°) than did females (17° ± 6°), in what the authors hypothesized may be an effort to compensate for decreased hip and shoulder rotation.

#### Lead Knee Axial Rotation

In their study of a single subject with a lead knee TKA, Hamai et al. [39] used video-fluoroscopy to measure the relative axial rotation of the tibial and femoral components, with the following measurements: address −6.3°, early-BS −7.4°, late-BS −8.1°, top-BS −13°, and end-FT −2.7°. Positive values indicate internal rotation of the tibia [39]. Using skin marker-based motion capture, Somjarod et al. [50] reported significant differences in lead knee axial rotation between two professional and two amateur players at the mid-DS (−15° ± 5° vs. −8° ± 2°), impact (2° ± 2° vs. 10° ± 3°), and mid-FT (4° ± 2° vs. 11° ± 4°) phases.

#### Trail Knee Flexion/Extension

**Fig. 4:**
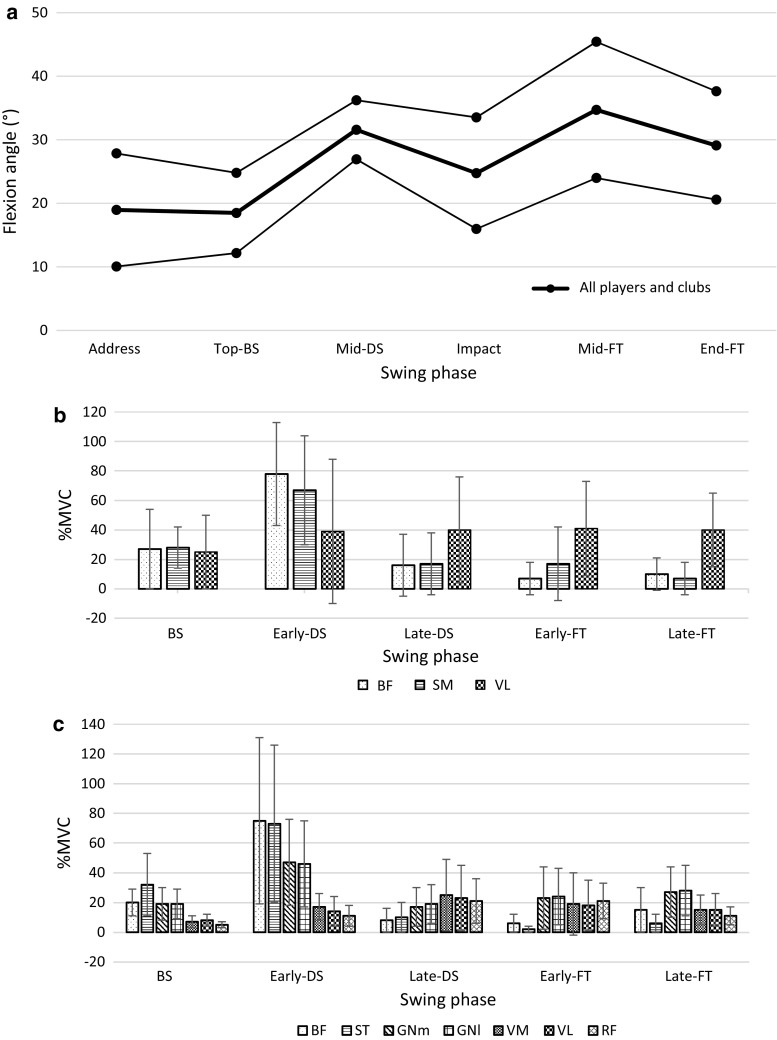
**a** Trail knee flexion angle of players of all skill levels using all club types at the six major swing phases: address, top of the backswing (top-BS), middle of the downswing (mid-DS), impact, middle of the follow-through (mid-FT), and end of the FT (End-FT). Thick lines and thin lines represent the combined mean and standard deviation of all study groups, respectively [39–41, 44, 45, 47, 49]. Electromyographic activity (mean ± standard deviation) as a percentage of muscle activity during maximum voluntary contraction (%MVC) of muscles crossing the knee joint at five phases of the golf swing: BS, early-DS, late-DS, early-FT, and late-FT, reported by **b** Bechler et al. [53] and **c** Marta et al. [54]. Muscles analysed: biceps femoris (BF), semimembranosus (SM), vastus lateralis (VL), semitendinosus (ST), gastrocnemius medialis (GNm), gastrocnemius lateralis (GNl), vastus medialis (VM), rectus femoris (RF)

Seven studies also included results for trail knee kinematics [39–41, 44, 45, 47, 49]. The trail knee in the sagittal plane exhibited a smaller range of flexion as well as less rapid movements throughout the course of the swing compared with the lead knee (Fig. 4). No significant differences were found when comparing the angular velocity of the trail leg between groups with high and low ball velocity [46]. The results presented by Somjarod et al. [50] also showed that maximum trail knee angular velocity occurred during the mid-DS phase for both professionals and amateurs, but the magnitude was far less than that of the lead knee (137.8 ± 31.4 vs. 113.1 ± 10.6 deg/s) [50].

#### Trail Knee Axial Rotation

The axial rotation of the trailing leg was measured in three subjects with a TKA using video-fluoroscopy [39]: address 9.8° ± 7.7°, early-BS 12.5° ± 7.6°, late-BS 13.9° ± 6.6°, top-BS 16.0° ± 6.7°, and end-FT −5.5° ± 4.9°. Using skin marker-based motion capture, Somjarod et al. [50] also reported axial rotations of the trail knee: early-BS −3.0° ± 4.1° vs. −2.2° ± 1.9°, mid-BS 2.3° ± 4.0° vs. 6.8° ± 1.0°, top-BS 4.5° ± 3.8° vs. 9.4° ± 1.5°, mid-DS −13.5° ± 1.9° vs. −8.0° ± 2.7°, impact −13.4° ± 2.2° vs. −9.9° ± 2.9°, and mid-FT 12.3° ± 2.4° vs. −9.0° ± 4.2°.

Finally, a study using skin-mounted markers to assess older men found that both lead knee peak internal (20° ± 7°) and external (14° ± 5°) rotations exceeded those of the trail knee (15° ± 6° and 10° ± 6°, respectively) [48]. The effect of club influence on knee axial rotation has not yet been reported in the literature.

### Kinetics

The literature search identified six studies that calculated or measured the forces and/or moments occurring at the knee during the golf swing [7, 10, 11, 44, 48, 51]. Four studies used inverse dynamics driven by motion capture and ground reaction forces to calculate the external moments and reaction forces [7, 44, 48, 51]. The remaining two studies reported results measured from subjects with instrumented TKAs [10, 11].

#### Forces

The peak compressive force calculated using inverse dynamics was 100.0 ± 18.9%BW in the lead knee and 71.5 ± 8.7%BW in the trail knee, occurring at 29.5° ± 9.2° and 21.5° ± 6.0° of flexion, respectively [7] (Table 3). Contrary to these early results, D’Lima et al. [11] measured tibio-femoral contact forces in the lead knee of up to 440 and 320%BW in the trailing knee. Additionally, the difference between lead knee contact force when using a sand wedge and driver was only 30%BW [11] (Table 3). A second study measuring a single left-handed player with a right (lead) knee instrumented implant reported contact forces of 320%BW occurring at 27–30° of flexion [10] (Table 3).

**Table 3:** Knee joint contact forces as a percentage of bodyweight during golf reported in the literature

Study	Condition/anatomical direction	Lead knee (%BW)	Trail knee (%BW)	Unspecified knee (%BW)
Gatt et al. [7]. 13 men, age 35 ± 14.2 y, mean HC 11.2 (4–18), inverse dynamics approach, 5-iron	Compressive	99.9 ± 18.9	71.5 ± 8.7	
	Anterior	39.0 ± 10.7	19.9 ± 5.0	
	Posterior	−0.3 ± 2.6	10.1 ± 3.5	
	Medial	9.9 ± 3.3	9.5 ± 2.8	
	Lateral	17.0 ± 8.6	11.4 ± 4.2	
D’Lima et al. [11]. Two men aged 83 and 81 y, one woman aged 67 y; HC not reported; instrumented knee implant, driver and sand wedge	Driver: compressive	440	320	
	Sand wedge: compressive	410		
	Driver: anterior shear			34 ± 1
Mündermann et al. [10]. One man aged 81 y, HC not reported, right instrumented knee implant, club unspecified, handedness obtained from author correspondence	Compressive	320		

Values are presented as mean ± standard deviation (range) unless otherwise indicated

*HC* handicap, *y* year, *%BW* percentage of bodyweight

Although only a few quantitative results have been presented, anterior–posterior shear forces in the lead knee calculated using inverse dynamics [7] suggested magnitudes in a range comparable to that measured using instrumented implants [11]: 39 ± 11 and 34 ± 1%BW, respectively; knee unspecified (Table 3).

#### Moments

Only two studies reported the magnitude of both abduction and adduction moments calculated using inverse dynamics [7, 51]. Lynn and Noffal [51] reported abduction moments with the lead foot straight at address and externally rotated by 30° that were similar in magnitude to those published by Pfeiffer et al. [48] (Table S1 in the Electronic Supplementary Material [ESM]). However, this external rotation of the lead foot did significantly reduce the magnitude of adduction moments when compared with the straight foot stance [51]. Conversely, Gatt et al. [7] calculated larger adduction than abduction moments in the lead knee (Table S1 in the ESM). The comparative magnitude of flexion and extension moments in the lead knee was inconsistent across studies. Flexion moments ranged from 0.10 [44] to 1.26 ± 0.41 Nm/kg [7], whereas extension moments ranged from 0.27 ± 0.31 [7] to 1.15 Nm/kg [44] (Table S1 in the ESM) [7, 11, 44, 48, 51]. The magnitude of knee axial rotation moments has only been reported twice in the literature, once calculated using inverse dynamics and once using instrumented TKAs [7, 11]. Although D’Lima et al. [11] did not indicate the direction of the measured axial moment or the knee in which it was measured, the magnitude (0.17 ± 0.02 Nm/kg) was comparable to that reported by Gatt et al. [7] for lead knee internal rotation (0.21 ± 0.07 Nm/kg) but less than the external rotation moment (0.36 ± 0.13 Nm/kg). Additionally, Gatt et al. [7] calculated significantly smaller magnitudes of both internal and external rotation moments in the trailing knee when compared with the lead knee.

Magnitudes of flexion and extension moments about the trail knee during the golf swing were within similar ranges across studies [7, 44, 48] (Table S2 in the ESM). Choi et al. [44] observed that less skilled golfers exhibited a more random pattern of peak knee flexion moment in relation to knee flexion angle than did their more consistent skilled counterparts.

### Electromyography

Three studies reporting muscle activity about the knee during the golf swing were identified. Carlsöö [52] measured over 300 5-iron swings from a single professional male golfer, but little detail was provided as to the data-collection methods used, and only qualitative data could be extracted from the results. Bechler et al. [53] utilized fine wire insulated needles inserted directly into muscle bellies to measure three muscles crossing the knee joint (biceps femoris [long head], semimembranosus, and vastus lateralis) during the driver swings of 13 skilled amateur golfers. More recently, surface electrodes were used to measure the activity of six muscles crossing the knee joint (vastus medialis, vastus lateralis, rectus femoris, biceps femoris, semitendinosus, gastrocnemius medialis, and gastrocnemius lateralis) during the swings of players using a pitching wedge, as well as a 7- and 4-iron [54]. The two latter studies expressed muscle activity as a percentage of a maximum muscle voluntary contraction (%MVC).

#### Lead Leg

Qualitative assessment of results presented by Carlsöö [52] showed that, following top-BS, the flexors of the lead leg (biceps femoris, semimembranosus, and semitendinosus) experience maximum activation, which is maintained until the late-FT. At early-DS, the major extensors of the knee (rectus femoris, vastus medialis, and vastus lateralis) all experience an increase in activity, reaching a peak around impact. Following impact, the biceps femoris, semimembranosus, and semitendinosus muscles remain activated during the early-FT, followed by a decrease in activation until late-FT. Concurrently, the activity of the rectus femoris, vastus lateralis, and vastus medialis remains moderate immediately following impact and gradually decreases as the FT continues to the finish of the swing [52].

Bechler et al. [53] measured high levels of activation in the vastus lateralis (88%MVC) during the forward swing, which was maintained into the early-FT (59%MVC). Activity of the biceps femoris and semimembranosus also peaked during phases of the forward swing, with activation levels of 83 and 51%MVC, respectively (Fig. 2). Similar results reported by Marta et al. [54] showed high levels of quadriceps (vastus medialis, rectus femoris, and vastus lateralis) activity, i.e., 43–58%MVC during the forward swing. Muscles of the hamstrings (biceps femoris and semitendinosus) also showed peak activity of 33–57%MVC during the latter stages of the forward swing (Fig. 2). Additionally, no significant difference in lead leg muscle activity was reported between the use of a pitching wedge, a 7-iron, and a 4-iron [54].

#### Trail Leg

During the backswing, all three studies measured minimal activity in the extensor muscles but moderate levels of activity in the flexors of the trail knee. Most notably, all three reports measured peak knee flexor activity during the early stages of the DS, which was immediately followed by a major decrease in activation prior to impact [52–54]. Extensors of the trail leg showed consistent activation throughout all phases of the forward swing and FT, but the magnitude of peak activity was less than that of the knee flexors [53, 54] (Fig. 4). Marta et al. [54] reported significant differences in activation levels between the 4-iron and pitching wedge in all muscles besides the vastus lateralis [53].

## Discussion

Golf is considered a low-impact sport; however, surveys have shown that knee injuries do occur as a result of participation. This systematic review of the literature revealed that the prevalence of knee injury generally ranges from 3 to 18%, demonstrating a prevalence comparable to that of high-impact sports such as basketball [55–58]. Consensus within the literature indicates that most golf injuries occur as a result of either overuse or poor and inconsistent technique [6], and indeed these were found to be the two most frequently cited causes for injury of the knee in both amateur and professional cohorts [5, 17]. Two of the highest knee injury rates reported in the literature referred to groups of professional or highly proficient amateur golfers [16, 18], with senior professional golfers surveyed by Sugaya et al. [16] demonstrating a knee injury rate of 15%. Additionally, McCarroll and Gioe [14] found that 68.7% of professional golfers’ injuries were due to repetitive practice swings. Similarly, some studies have shown that older amateur players are at greater risk of knee injury during golf than are younger players [19, 27, 28], whereas other results indicate older players are more likely to aggravate a previous condition [17]. This review indicates that older players most likely experience a higher prevalence of knee injuries; however, data are insufficient to conclude that these injuries resulted only from participation in golf. Still, these results may suggest that the combined loading conditions at the knee associated with both a skilled player’s and an amateur’s golf swing are sufficient to result in progressive damage of the joint, re-aggravation of a previous condition, and possibly eventual traumatic injury.

The notion that players may be prone to trauma through aggravation of a previous injury is consistent with the results of multiple studies [5, 17, 29]. In fact, Gosheger et al. [5] reported that 31.3% of players experiencing chronic knee pain prior to golf felt that playing the game had worsened their symptoms. Additionally, Guten [29] found that 15 of 35 golfers reporting to a clinic with knee injury had previously undergone meniscectomy. These results indicate that players are likely at risk of injury due to differing mechanisms; however, these may be influenced by their previous history of knee injury, level of participation, and for how long they have played golf.

As most studies focus on the more prevalent older cohorts, the effects of playing and practicing golf at a younger age have not been well documented. According to Cabri et al. [6], younger players are rarely exposed to overuse conditions that are conducive to musculoskeletal injury; however, this statement may not be as relevant as it once was. Given the emergence in recent years of intensive programs to cater to players aged <18 years [59, 60], it would be reasonable to assume that some of these younger participants, particularly young elite, may be subject to repetitive overuse loading conditions. Apart from age, no clear consensus was reached among studies in identifying subject groups at high risk of knee injury. Surveys of both male and female groups indicated little consistent evidence of injury bias, regardless of skill level [14, 16–18, 61]. Similarly, there was little difference in the rate of injury between professionals and amateurs across studies (Table 1).

Golf is commonly used as part of post-operative rehabilitation programs. In contrast, high-impact sports such as jogging and singles tennis are often not recommended or allowed [3, 4, 62–66], with such advice based mainly on the clinician’s experience and self-assessment of the patient’s condition [1, 3, 4, 65]. Although the number of instances where larger loads may be experienced during a game of golf will be far less than in many other activities, loads reported by instrumented implant studies have shown the golf swing has the potential to generate loads in the lead knee slightly higher than those during tennis and consistent with those during jogging [11]. Mallon and Callaghan [3] showed that a large number of players, especially those with lead knee TKAs, experienced pain during and after golf. These data suggest a difference in the internal loading conditions between the lead and trail knees, which would be in accordance with biomechanical concepts as well as measured joint contact forces and ground reaction torques, which suggest consistently greater magnitudes in the lead leg [11, 67, 68]. Interestingly, a non-peer-reviewed informal survey conducted by former Professional Golfers’ Association (PGA) Tour and Champions Tour professional Howard Twitty [69] found that 55% of players interviewed at a 2009 PGA Champions Tour (50+ age group) event had experienced a knee injury at some point in their career—possibly indicating an association between long-term golfing participation and injury. In his informal survey, Twitty also reported that 83% of injuries were to the left knee, and only 17% occurred in the right knee [69]. Although the handedness of the players was not specified, it could generally be assumed that the majority of golfers are right handed and that these injuries therefore occurred in the lead (left) knee.

Multiple surveys have reported that rates of return to golf following TKA range from 30 to 57% [63, 64, 70], although patients who have received unicompartmental arthroplasty seem to have higher rates of return [64, 71]. Pain and discomfort may account for the disparity between the apparent lack of restrictions regarding return to golf following TKA and the often low rates of return that actually occur [63, 64, 70]. Such a conclusion is supported by the fact that TKA golfers experience greater pain than age-matched controls who do not play [33]. Return to golf following TKA also seems to be an individualized issue, as some aspects of the game, such as driving from the tee, became relatively easier or harder for different players post-TKA [72]. However, it must also be taken into consideration that the ease with which players can perform certain shots may be highly dependent on the knee that was injured or replaced, the golfer’s technique, or even the specific implant and its interaction with the surrounding soft-tissue structures. As a result of these aforementioned factors, it seems that clinicians should take into account the laterality of a TKA when advising return to golf. Additionally, it seems that there is a need for further investigation into the potential risks golf poses to TKA damage in order to better inform clinicians.

Recommendations regarding injury prevention by means of technique changes or use of equipment have also lacked supporting evidence. The use of shorter clubs has often been recommended to reduce the intensity of the swing and therefore the loads on the knee [8, 29, 73]. However, D’Lima et al. [11] found no significant difference between the compressive loads in the knee when using a driver as opposed to a sand wedge. Additionally, the amount of torque generated at the ground by the lead foot has been found to be similar, regardless of the club used [67, 68]. Spike-less shoes have also been suggested as a potential method to reduce the torque experienced in the knee [29, 72], but Gatt et al. [7] found no significant influence on mean peak forces and moments in the lead knee with or without them. Similarly, Worsfold et al. [67, 68] reported no significant difference in lead foot torque generation when using classic spikes, modern spikes, or flat-soled shoes, indicating that this commonly advised equipment change may not decrease the risk of knee injury due to excessive torsion at the knee joint [7].

These results indicate that such common recommendations to reduce knee loading through equipment changes, and thereby reduce injury risk, lack supporting evidence. Similarly, few studies have examined the efficacy of technique changes in decreasing a player’s risk of knee injury. Specifically, the influence of flexion angle and weight distribution on loading conditions at the knee remains inconclusive [29, 73, 74], However, the inconsistency in flexion and extension moments across kinetic studies demonstrates that loading patterns obviously differ between players. Given the magnitude of these moments is likely influenced by the knee flexion angle throughout the swing, technique changes may aid in reducing these external moments experienced by the knee. The common recommendation to externally rotate the lead foot by 30° has indeed been shown to result in significant reduction of external adduction moments in the lead knee [51]. Here, a wide range of factors, including age, skill level, technique, physical strength, flexibility, warm-up habits, etc. clearly vary from player to player. Therefore, general recommendations regarding technique, especially in subjects returning from knee injury or TKA, may not be valid for all golfers, and a more individual analysis that considers their specific biomechanical circumstances might aid in a reduction of injury risk.

Although a common pattern of knee motion was evident in golfers’ swings using most clubs, it is clear that the magnitude of knee flexion and axial rotation can vary greatly between individuals and appears to be dependent on technique rather than skill level. However, the rate of extension in the lead knee during the DS was shown to be greater in players with a higher ball velocity, an attribute associated with most professionals [46, 50]. The magnitudes of knee axial rotation at each phase of the swing varied between subjects; however, a clear pattern of tibial external rotation during phases of the BS followed by a large amount of internal tibial rotation during the forward swing and following impact was observed [39, 50]. The different magnitudes of tibial internal rotation could possibly be due to the error associated with skin marker kinematic measurement when compared with fluoroscopic measurement [75]. Alternatively, the comparison of professional and amateur players by Hamai et al. [39] suggests this may be another example of the variance in kinematics that would result from differing techniques. Regardless, it was shown that, in TKA golfers, the ability to “rapidly generate unusual magnitudes of axial rotation,” combined with the large range of motion (average 18.7° from the top of the BS through to impact) [39], may raise concerns that the golf swing could be detrimental to implant health. Here, the range of knee rotation could result in contact locations at the edges of the polyethylene surface, potentially leading to chronic wear and implant damage [39].

Although only representative of a single TKA subject, Mündermann et al. [10] found that an amateur golf swing was able to generate a loading pattern where a greater proportion of the peak load was placed on the lateral compartment than the medial, a pattern that was not seen in any measured activity of daily living. These results, along with reports that 54% of all TKA golfers assessed, and 79% of those with cemented implants, had experienced radiographic loosening, all indicate that lead knee kinematic and kinetic factors associated with certain techniques may place undue stresses on implant components and plausibly also structures of a natural knee [3]. The anterior cruciate ligament (ACL), ligamentous structures of the medial and lateral knee as well as the posterior medial capsule have been shown to play a crucial role in resisting internal tibial rotation, especially at flexion angles lower than 30° [76–82]. Given the kinematic patterns, and particularly the high levels of tibio-femoral torsion, associated with the golf swing, it is possible that these structures are exposed to strains that may not be experienced during activities of daily living and might therefore be at increased risk of injury.

A key factor in establishing potential injury mechanisms and therefore risk of injury is the loads experienced by the knee joint during a movement. Only two studies reported both the kinematics and the kinetics of the knee during golf. The flexion angle at peak compressive load in the lead knee reported by Gatt et al. [7] and by Mündermann et al. [10] was 29.5° ± 9.2° vs. 27°–30°, respectively. However, Gatt et al. [7] concluded there was no discernible consistent loading pattern during the golf swing, due to the large inter-subject variability of the mean peak forces and moments, together with the knee alignment when these loads occurred. However, the magnitude of peak forces differed significantly between those measured in subjects with instrumented TKAs (lead 320–440%BW and trail 320%BW) [10, 53], and those reported by Gatt et al. [7] (lead 99.9 ± 18.9%BW and trail 71.5 ± 8.7%BW). These results highlight the importance of the additional forces due to muscle activity that are not considered in inverse dynamics approaches. Consequently, the conclusions of reviews based on the resultant forces from the inverse dynamics analysis of Gatt et al. [7], rather than the “bone-on-bone” joint contact forces that are known to occur in vivo, should therefore be interpreted with caution [6, 8, 83].

Although only three studies were found that reported muscular activity about the knee during golf, common trends in muscle activation of both the lead and the trail legs were identified. Trail knee muscles become largely inactive once the majority of body weight has been transferred to the lead leg, following the early stages of the DS. However, both flexor and extensor muscles crossing the lead knee show high levels of activation from the top of the BS onwards. This suggests that co-contraction is used to stabilize the knee joint, possibly explaining the high tibio-femoral joint contact forces (1943 N) measured in an instrumented knee TKA at the corresponding vertical ground reaction force of only 340 N [10].

Finally, the effects of fatigue should also be considered when attempting to identify risk factors for knee injury during golf. Golfers will often choose to walk the length of the course during play, which can involve crossing uneven terrain, especially off the fairway. Vandervoort et al. [84] noted that walking associated with golf provides an opportunity to maintain some level of cardiovascular fitness; however, over the course of a match, this may contribute to increased fatigue, especially in older players. As a result, it is possible that the knee may be less equipped to balance the external forces occurring during a swing. Although not extensively investigated in the literature, unusual or awkward lies may also result in a stance and swing that produces more demanding kinematics and greater knee loads. Such conditions could increase the risk of knee injury, especially if a player is also experiencing fatigue of the lower limb muscles. Therefore, in some cases, it may be beneficial for certain players to avoid walking long distances while playing golf and/or to avoid playing unconventional and awkward shots.

This review seems to indicate that, contrary to the perceived low-load/low-injury risk impression given to players, a complex set of conditions occur in the knee during the forward swing that could result in injury. Given the lack of published results detailing the specifics of injury mechanisms that would account for the injury rates reported in the literature, an analysis of the kinematics and kinetics experienced by the knee joint during the golf swing may aid in identifying potential structures that are subject to stress. This review has identified the following patterns of motion and loading that may be experienced by the knee, especially on the leading side, during a golf swing:

- rapid knee extension occurring at a range of joint flexion between 0° and 30°,
- considerable internal tibial rotation and large ground reaction axial torque,
- at low flexion angles, hamstring activity is ineffective in actively restraining anterior tibial displacement and therefore mainly contributes towards greater compression of the joint,
- strong quadriceps activity contributing to high joint loading,
- due to the natural anterior to posterior slope of the tibial plateau, compression of the tibio-femoral joint (resulting from the aforementioned muscle forces) produces anterior tibial displacement.

This combination of joint kinematics and kinetics would suggest that structures of the knee resisting joint compression and internal rotation of the tibia in a knee joint flexed at ≤30° (a common sports injury mechanism) may be susceptible to injury [75–77, 79, 85–88]. As a result, ACL rupture [77, 89–91], chondral and osteochondral injuries to both the femoral condyles and tibial plateau, as well as associated injuries to the menisci and/or posterior-medial capsule, are potential injuries that may occur from undue and repetitive stress resulting from the swing [80, 86–88, 92]. Furthermore, excessive axial rotation can result in contact between the femoral condyles and the tibial spine, where the cartilage is less robust and may therefore be susceptible to damage at lower magnitudes of load [86, 88]. Importantly, such injuries could occur as a result of sudden trauma or repetitive loading, both of which have been identified as possible mechanisms for knee injury during golf. Indeed, evidence of injuries to some of the aforementioned structures has been seen in some case reports [29, 36]. Although it is often difficult to attribute the occurrence of many injuries purely to involvement in golf as opposed to other activities, this biomechanical analysis may help to explain the mechanisms responsible for undiagnosed injuries reported in the literature to date. While it is evident that this combination of conditions could present a risk of knee injury, especially to subjects returning from previous injury or TKA, it is clear that the knee is normally capable of withstanding the loads generated during the golf swing. Although further investigation into these injury mechanisms and their relationship with the golf swing are critically required, clinicians and coaches should consider each specific patient’s participation in golf, especially taking into account the laterality of the injured knee.

This review is not without limitations. For example, retrospective studies included in the review are inherently subject to recall bias and, as a result, the statistics of injury rates and, if addressed, the factors that contributed to the cause or irritation of such injuries, may not be entirely accurate [23]. Another drawback associated with survey results such as these is the fact that it is often difficult to attribute the occurrence of many injuries purely to involvement in golf as opposed to other activities. However, the greatest limitation regarding the interpretation of knee injury statistics is the lack of information surrounding the nature of the injury recorded, given many studies did not enquire as to the laterality, diagnosis, or etiological basis of the injury.

Some limitations were also present when reviewing knee kinematics. First, all studies were performed in either a driving range or laboratory environment with level ground and ideal lie conditions. Greater variability in knee kinematics will almost certainly be present during real play on a course with a variety of terrains. Here, skill level may have a greater impact on a player’s ability to execute consistent motions with a changing position relative to the ball, a factor that is yet to be considered in studies reporting knee kinematics. Second, all but one study measured knee kinematics using optical motion capture techniques, an approach that is inherently affected by soft tissue artefact [93], especially when assessing axial rotation, which has been identified as a potential factor in the mechanism of knee injury during golf. Lastly, heterogeneity in study designs when measuring kinematics makes comparisons between specific groups difficult, also resulting in differing definitions of swing phases where knee flexion angles were reported. Despite these issues, this systematic review of the literature now represents the state-of-knowledge regarding risk factors for knee injury in golf.

## Conclusion

This systematic review of the literature indicates that injuries to the knee account for 3–18% of all golf-related injuries. To date, the majority of studies addressing knee loading in golf have based conclusions on the results of a single inverse dynamics study that underestimated the true magnitude of the in vivo joint contact forces as measured in instrumented implants. This review is therefore the first to identify this inconsistency and provide a clear, informed overview of the rates of knee injury in golf, the loads that occur in the knee, the associated joint kinematics, and the injury risks.

Details surrounding the laterality, mechanisms, and type of knee injuries that players experience are scarce. However, the literature reports loads generated in the knee during the golf swing (320–440%BW) [10, 11] that are in excess of some activities of daily living (level walking 261%BW, squatting 253%BW, stair ascent 316 %BW, stair descent 346%BW) [94] and comparable to some higher-intensity activities (tennis serving 424%BW, jogging 439%BW) [11]. These loads alone are therefore unlikely to be of a magnitude that poses a high risk of traumatic injury. However, the addition of tibial internal rotation at low flexion angles is likely to expose the knee, especially on the leading side, to more aggressive conditions. As a result, it seems reasonable that the loading conditions occurring during the golf swing could contribute towards repetitive degeneration and overuse injuries. Moreover, players with a prior history of knee injury or after total knee replacement might plausibly be exposed to a risk of more serious traumatic injury.

Although injury reports are yet to definitively establish which knee has a higher rate of injury, the lead knee appears to be exposed to higher magnitudes of stress and more complex kinematics than the trailing knee. Recommendations regarding return to golf following knee injury or surgical intervention should carefully consider the laterality of the injury. Currently, very few of the modifications to golfing equipment or technique suggested in the literature are based on empirical evidence; however, it is possible that avoiding awkward lies that may require an unconventional swing could help avoid placing unnecessary stress on a player’s knees. Additionally, fatigue associated with walking long distances during golf may also reduce the knee’s ability to deal with external loads and motions during the swing, thereby increasing risk of injury.

In light of this, and given the importance of golf for many players with respect to remaining active, fit, and social, further research aimed at identifying potential loads associated with an individual’s swing, as well as situations that may increase stress on the knee joint such as uneven or awkward lies, technique, or fatigue, should be pursued. This will better inform the clinician, golf professionals, and players alike about reducing risk of knee injury. Until such time, the status quo that golf poses little risk of injury should be reconsidered, especially for those who have experienced previous knee joint damage.

## Electronic supplementary material

Below is the link to the electronic supplementary material.

Supplementary material 1 (DOCX 17 kb)

Supplementary material 2 (DOCX 16 kb)
