# Supplementary material for: Risk Factors for Knee Injury in Golf: A Systematic Review
Source: Sports Med. 2017 Sep 7;47(12):2621–39. doi: 10.1007/s40279-017-0780-5 (PMC5684267; doi:10.1007/s40279-017-0780-5)
Supplement: Supplementary file 2 — Supplementary material 2 (DOCX 16 kb) [file 40279_2017_780_MOESM2_ESM.docx]

| Club | Study | Condition/ subject | Flexion | Extension | Abduction | Adduction | Internal Rotation | External Rotation | Additional details |
| --- | --- | --- | --- | --- | --- | --- | --- | --- | --- |
| Driver | Pfeiffer et al., 2014 [48] 11 men, age: 57.7±8.5y, AS: 82.8±6.8 inverse dynamics | Trail leg | 0.58±0.25 |  | 0.48±0.14 |  |  |  |  |
|  | Choi et al., 2015 [44] Professionals: 12 men, 6 women, age: 28.6±8.3y  Amateurs: 18 men, 5 women, age: 42.8±10.1y, HC: 18.5±7.4, inverse dynamics | Professional 1 | 0.70 | 0.89 |  |  |  |  |  |
|  |  | Professional 2 | 0.60 | 1.10 |  |  |  |  |  |
|  |  | Amateur 1 | 0.77 | 0.89 |  |  |  |  |  |
|  |  | Amateur 2 | 0.90 | 0.22 |  |  |  |  |  |
| 5-iron | Gatt et al., 1998 [7] 13 men, age: 35±14.2y, HC: mean 11.2 (4-18) inverse dynamics | Trail Leg | 0.76±0.21* | 0.89±0.21* | 0.51±0.23* | 0.69±0.24* | 0.25±0.08* | 0.26±0.11** | * significantly different from lead leg p<0.001 ** significantly different from lead leg p<0.05 |
| Unspecified | D’Lima et al., 2008 [11] 2 men: 83 and 81y 1 woman: 67y instrumented knee implant |  |  |  |  |  | 0.17±0.02 | | Direction unspecified |

**Electronic Supplementary Table S2:** Trail knee moments (Nm/kg) during the golf swing reported in the literature. Results presented by D’lima et al. are those measured internally using an instrumented TKA, all other moments from inverse dynamics based studies are external moments. HC= handicap, AS= average score, values presented are given as mean ± SD (range). The knee in which axial rotation moments were measured by D’Lima et al. was not clearly specified, therefore these results are considered for both the lead and trailing knees.
